# GMP-compliant, serum-free cultures preserve therapeutic potential of extracellular vesicles from human mesenchymal stromal cells

**DOI:** 10.3389/fcell.2025.1633912

**Published:** 2025-09-01

**Authors:** Filippo Calascibetta, Annalisa Martorana, Margot Lo Pinto, Claudia Carcione, Salvatore D’Arpa, Giandomenico Amico, Vitale Miceli, Nicola Cuscino, Gioacchin Iannolo, Lorenzo Volpe, Simone Dario Scilabra, Pier Giulio Conaldi, Cinzia Maria Chinnici

**Affiliations:** ^1^ Regenerative Medicine Unit, Ri.MED Foundation, Palermo, Italy; ^2^ Proteomics Group, Ri.MED Foundation, Palermo, Italy; ^3^ Plastic Surgery and Breast Unit, La Maddalena Clinic for Cancer, Palermo, Italy; ^4^ Department of Research, IRCCS ISMETT, Palermo, Italy; ^5^ Department of Medicine and Surgery, Kore University, Enna, Italy

**Keywords:** mesenchymal stromal cells, extracellular vesicles, GMP-compliant medium, liver fibrosis, LX-2 cells, collagen secretion

## Abstract

The therapeutic potential of extracellular vesicles (EVs) derived from human mesenchymal stromal cells (MSCs) is limited by the lack of standardized, Good Manufacturing Practice (GMP)-compliant production protocols. This study investigates the effects of MSC-Brew, a commercially available GMP-grade medium, on MSC-derived EVs in comparison to those produced in conventional cultures with DMEM supplemented with 10% fetal bovine serum (FBS). MSCs from adult dermis were successfully isolated and expanded in Brew medium while retaining their characteristic surface marker expression. MSC-EVs derived from Brew cultures met the Minimal Information for Studies of Extracellular Vesicles (MISEV) criteria, including particle size, concentration, marker expression, and minimal inflammatory cytokine content. Notably, Brew-EVs exhibited a significantly higher particle-to-protein ratio compared to EVs produced in FBS-containing cultures, indicating improved purity. Proteomic analysis revealed a largely conserved composition between Brew-EVs and conventionally produced EVs, and microRNA (miRNA) profiling identified only four differentially expressed miRNAs. Brew-EVs were enriched in anti-fibrotic miRNAs and effectively reduced collagen secretion in transforming growth factor (TGF)-β1-activated LX-2 cells, a human hepatic stellate cell line used as a model of liver fibrosis. These findings support MSC-Brew medium as a standardized, serum-free platform for the consistent production of high-quality EVs suitable for therapeutic applications.

## 1 Introduction

Extracellular vesicles (EVs) can originate from various cell types, including mesenchymal stromal cells (MSCs), T cells, natural killer (NK) cells, and tumor cells. The cellular source of EVs plays a critical role in determining their molecular cargo and biological function, making source selection a key consideration in the development of EV-based strategies for therapeutics, drug delivery, and diagnostics. Therapeutic applications have primarily focused on EVs derived from immune cells or MSCs. For example, autologous immune cell-derived EVs have shown promise in modulating immune responses in a targeted manner. MSC-derived EVs are known to deliver regulatory microRNAs (miRNAs), such as miR-155 and miR-146, which modulate innate immune signaling and vesicle trafficking, resulting in the suppression of T, B, and NK cell activity. In murine models, MSC-EVs enriched in CD73^+^ and CD34^+^ subpopulations have demonstrated the ability to reverse graft-versus-host disease (GvHD) and support recovery from hindlimb ischemia by attenuating Th1-driven inflammation (Mann et al., 2022). In contrast, EVs derived from tumor cells are not used as therapeutics; instead, they are being explored as diagnostic biomarkers and therapeutic targets in oncology. Tumor-derived EVs carry tumor-associated antigens and immunomodulatory molecules that can influence the tumor microenvironment and immune response. Their ability to present tumor antigens has made them valuable in the development of cancer immunotherapies, particularly as targets for immune recognition and as carriers for drug delivery (Raghav and Mann, 2021).

Human MSC-EVs have emerged as promising therapeutic agents due to their ability to deliver bioactive molecules that promote anti-inflammatory, anti-fibrotic, and pro-regenerative effects, while maintaining a favorable safety profile (Liang et al., 2019; Kou et al., 2022; Zhao et al., 2023). In addition, MSC-EVs show great potential as off-the-shelf therapies targeting rapid wound healing, cartilage injuries, osteoarthritis, and related conditions (Raghav et al., 2022; Raghav and Mann, 2024). These findings highlight the promising translational applications of MSC-EVs in regenerative medicine. Despite their therapeutic potential, the clinical translation of MSC-EVs faces significant challenges, including the lack of standardized manufacturing processes, batch-to-batch variability, and undefined release criteria. These factors complicate efforts to ensure product consistency, efficacy, and safety. Additionally, the unclear mechanisms of action (MoA) and limited pharmaceutical characterization of MSC-EVs further hinder regulatory approval and widespread clinical application (Rohde et al., 2019). Establishing standardized protocols for MSC-EV isolation, characterization, and quality control, particularly defining robust release criteria for purity, identity, and potency, is essential to overcoming these barriers.

Significant progress has been made in developing standardized MSC-EV manufacturing processes and adopting Good Manufacturing Practice (GMP) to improve reproducibility, scalability, and regulatory compliance (Rohde et al., 2019; Lau et al., 2022). A key aspect of this process is the selection of an appropriate culture medium that supports MSC isolation and expansion, while maintaining the quality and therapeutic efficacy of the EVs they produce. While several serum- and xeno-free media have been developed, MSC-Brew GMP Medium (Miltenyi) is among the few GMP-compliant options reported to support the isolation and expansion of MSCs from multiple tissue sources (Aussel et al., 2022). This highlights the potential for MSC-based therapies to be developed and manufactured under GMP conditions (Williams et al., 2025). However, these studies have primarily focused on MSC expansion, and the effects of Brew medium on EVs derived from MSCs remain unexplored. To our knowledge, this is the first study to evaluate the impact of MSC-Brew medium on both the characteristics and biological activity of EVs derived from adult dermis (AD)-MSCs. Notably, only one published study to date has examined MSC-EVs obtained under xeno-free, serum-free culture conditions (Chiabotto et al., 2024), underscoring the limited data available on EV production in clinically relevant settings.

MSCs were isolated and expanded using either MSC-Brew GMP Medium or standard Dulbecco’s Modified Eagle Medium (DMEM) supplemented with fetal bovine serum (FBS). EVs were subsequently isolated from the serum-free secretome via differential ultracentrifugation. EV assessment focused on key release parameters, including purity, identity, and biological activity. To ensure process consistency, we followed a published GMP-compliant workflow for MSC-EV production (Rohde et al., 2019). EV characterization was performed using Nanoparticle Tracking Analysis (NTA) in combination with Atomic Force Microscopy (AFM), as recommended by the Minimal Information for Studies of Extracellular Vesicles 2023 (MISEV2023) (Welsh et al., 2024). Additional assays included total protein quantification to evaluate purity, Western blot to confirm the presence of EV-associated markers, and Luminex-based cytokine profiling to assess inflammatory content as a safety parameter for therapeutic use. Beyond to essential release criteria, we also included “informative” tests, such as EV proteomic profiling and a disease-relevant evaluation of anti-fibrotic miRNA content.

To evaluate the biological activity of AD-MSC-EVs, we used a disease-relevant *in vitro* model based on immortalized hepatic stellate cells (LX-2; Xu et al., 2005) activated with the pro-fibrogenic transforming growth factor (TGF)-β1. Upon activation, LX-2 cells transdifferentiate into myofibroblast-like cells, characterized by increased collagen production and α-SMA expression (Böttcher and Pinzani, 2017). This model is widely used to assess the anti-fibrotic effects of therapeutic agents, including MSC-EVs (Dewidar et al., 2019; Bruno et al., 2020; Budi et al., 2021; Chiabotto et al., 2021). To enhance assay robustness, L-ascorbic acid (L-AA), a cofactor of prolyl 4-hydroxylases (P4Hs), was added alongside TGF-β1 during activation, as previously described (Smith-Cortinez et al., 2021). P4Hs are key enzymes involved in collagen biosynthesis (Myllyharju, 2003), and have been shown to be dysregulated in fibrotic conditions (Luo et al., 2015; Lin et al., 2021; Staab-Weijnitz, 2022; Zhang et al., 2023). Therefore, the addition of L-AA was intended to simulate pathological collagen deposition. As an anti-fibrotic treatment control, the pan-hydroxylase inhibitor dimethyloxalylglycine (DMOG) was also included. To our knowledge, this is the first study to apply the modified TGF-β1/L-AA activation protocol in LX-2 cells to evaluate the therapeutic potential of MSC-EVs, providing a more physiologically relevant model for assessing their anti-fibrotic activity.

## 2 Materials and methods

### 2.1 Isolation, culturing and banking of human MSCs from adult dermis

Human skin biopsies (15 × 15 cm^2^) were obtained from healthy adult donors (ages 20–40) undergoing plastic surgery, following approval by the Institutional Research Review Board (IRRB) of IRCCS ISMETT (IRRB/12/23) and after donors provided signed informed consent. MSCs were isolated from the dermis using a non-enzymatic cell outgrowth method (Chinnici et al., 2014; Iannolo et al., 2024). Briefly, biopsy fragments were placed dermis-side down onto treated 100 × 20 mm tissue culture dishes (Corning, Inc., Costar, NY, United States) and incubated at room temperature for 20 min. Culture medium, either MSC-Brew GMP medium (Miltenyi, Bergisch Gladbach, Germany) or high-glucose DMEM (Gibco, Thermo Fisher Scientific, Waltham, MA, United States) supplemented with 10% FBS (HyClone, Cytiva, Marlborough, MA, United States), glutamine, and antibiotic-antimycotic solution (AAS), was then added. The biopsies were maintained at 37°C with 5% CO_2_, and the medium was refreshed every 4 days. After 15 days, the outgrown cells were harvested using TrypLE Select (Gibco) and expanded in T75 flasks (passage 1) in their respective media conditions (Iannolo et al., 2024).

### 2.2 Immunophenotype analysis by flow cytometry

AD-MSCs from 10 donors, cultured in MSC-Brew GMP Medium or DMEM with 10% FBS, were analyzed at passages 2, 4, 6, and 8 for immunophenotypic markers, as previously described (Iannolo et al., 2024). Cells were washed with FACS buffer (PBS with 0.3% BSA and 0.1% NaN_3_; Sigma-Aldrich, St. Louis, MO, United States) and incubated on ice for 30 min with monoclonal antibodies against CD90, CD73, CD105, CD45, CD34, and HLA-DR (Becton Dickinson, Franklin Lakes, NJ, United States). After washing, cells were analyzed using a FACSCelesta™ flow cytometer (Becton Dickinson). The gating strategy included FSC/SSC selection, doublet exclusion (FSC-A vs. FSC-H), and viability assessment using 7-AAD (Becton Dickinson), as previously described (Busà et al., 2022). Data were analyzed with FlowJo software (FlowJo LLC, Ashland, OR, United States), with fluorescence intensity and marker expression evaluated relative to isotype controls.

### 2.3 Secretome collection and EV isolation

When AD-MSC cultures reached 80% confluence, the medium (MSC-Brew GMP or DMEM with 10% FBS) was removed, and the cells were washed three times with PBS. Serum-free alpha Minimum Essential Medium (α-MEM; Gibco, Thermo Fisher Scientific, Waltham, MA, United States) was then added, and the secretome was collected after 48 h. Initial debris removal was performed by centrifugation at 2,000 × g for 10 min (Palumbo et al., 2024). EVs (n = 6) were isolated from the secretome using differential ultracentrifugation, as previously described (Livshts et al., 2015), with an Optima XE-100 Ultracentrifuge (Beckman Coulter, Brea, CA, United States). The protocol included sequential spins at 20,000 × g for 40 min and 160,000 × g for 2 h, at 4°C.

### 2.4 EV characterization by NTA

Each EV pellet obtained by ultracentrifugation of 28 mL of secretome from AD-MSCs cultured in Brew medium (Brew-EVs) or standard DMEM with 10% FBS (DMEM-EVs) was resuspended in 125 µL of sterile PBS. The size distribution and concentration (particles per ml) of the AD-MSC-EVs were analyzed using a NanoSight NS3000 (Malvern Instruments Ltd., United Kingdom). For analysis, 500 µL of each diluted sample (1:100 in PBS) was injected into the instrument. Only EV preparations yielding 20 to 120 particles per frame were considered suitable for analysis, ensuring accurate size distribution and concentration measurements while minimizing errors from under- or over-saturation (Holcar et al., 2025).

### 2.5 EV characterization by AFM

The size and morphology of EVs was also assessed by AFM. Briefly, EV samples were prepared by drop-casting the EV solution onto a mica substrate, followed by vacuum drying. EV size was estimated by analyzing height profiles from multiple scans acquired at different locations on the mica substrate to ensure statistical significance. AFM imaging was performed using a FASTSCAN microscope (Bruker Corporation, Billerica, MA, United States) equipped with a closed-loop piezo scanner, as previously described (Chiappara et al., 2020), providing a maximum X-Y scan range of 35 µm and a Z range of 3 µm. Scans were collected in tapping mode in air, using a FastScan A probe with a nominal tip radius of 5 nm and a pixel resolution comparable to the tip radius.

### 2.6 Total protein extraction from MSCs and EVs

MSCs in a 6-well plate were lysed with 130 µL of ice-cold 1X radioimmunoprecipitation assay (RIPA) buffer (Pierce, Thermo Fisher Scientific, Northumberland, United Kingdom), supplemented with Halt Protease and Phosphatase Inhibitor Cocktail (Invitrogen, Breda, Netherlands). EV pellets obtained from ultracentrifugation of the secretome were resuspended in 80 µL of the same lysis buffer. Samples were incubated on ice for 15 min, followed by centrifugation at 14,000 × g for 15 min at 4°C. Total protein concentration was determined using the bicinchoninic acid (BCA) Protein Assay Kit (Pierce), with a Tecan Spark plate reader (Tecan, Männedorf, Switzerland).

### 2.7 Western blot analysis

Positive and negative EV markers were evaluated in both AD-MSCs and their EVs. The RIPA-extracted proteins were reduced in 4% Laemmli sample buffer (Bio-Rad Laboratories, Richmond, CA, United States) containing β-mercaptoethanol (Sigma-Aldrich). Briefly, 10 µg of protein per lane were separated on Mini-PROTEAN TGX precast 10% SDS-PAGE gels (Bio-Rad) and transferred onto polyvinylidene difluoride (PVDF) membranes (Bio-Rad) using the Trans-Blot Turbo transfer system (Bio-Rad). After blocking with 5% skim milk powder (Millipore, Darmstadt, Germany) in PBS containing 0.1% Tween-20 (PBS-T) for 1 h at room temperature, the membranes were washed three times with PBS-T and then incubated overnight at 4°C with primary antibodies diluted in PBS-T 0.5% BSA. The following antibodies were used: anti-Calnexin (C5C9, 1:1000; Cell Signaling, Danvers, MA, United States), anti-Alix (1:1000) (Cell Signaling), anti-CD81 (1:1000; Santa Cruz, Dallas, Texas, United States) and anti-β-actin (1:1000; Santa Cruz). Additionally, EV proteins were separated on Mini-PROTEAN TGX 4%–15% gradient gels (Bio-Rad) and incubated with anti-COL1A1 (1:1000; Invitrogen) and anti-fibronectin 1 (FN1) (1:1000; Abcam, Cambridge, United Kingdom) antibodies. After washing with PBS-T, the membranes were incubated with horseradish peroxidase (HRP)-conjugated secondary antibodies (anti-rabbit or anti-mouse IgG, 1:10,000; Promega), developed using Clarity Western ECL Substrate (Bio-Rad), and image capture was performed with the ChemiDoc XRS system (Bio-Rad).

### 2.8 Luminex-based quantification of inflammatory cytokine content

To quantify inflammatory cytokines, 50 µL of RIPA-extracted proteins from Brew- and DMEM-EVs (three samples per type) were directly loaded onto a 96-well plate pre-coated with the ProcartaPlex Human Inflammation panel (Invitrogen), which allows for the simultaneous detection of twenty cytokines. The analysis was performed using a Luminex-200 instrument (Luminex Corp., Austin, TX, United States), with dedicated software to measure protein concentrations in pg/mL. The resulting graph was generated using OriginPro version 8.5 (OriginLab Corporation, Northampton, MA, United States).

### 2.9 *Liquid chromatography with tandem mass spectrometry* (LC-MS/MS) proteomics of EVs and secretome

EV pellets (n = 3 per condition), collected by ultracentrifugation of the secretome and resuspended in 130 µL PBS, as well as 2 mL of secretome (n = 6 per condition), were processed using filter-aided sample preparation (FASP) with Vivacon 500 spin filters, 10 kDa cut-off (Sartorius, Göttingen, Germany) (Wiśniewski et al., 2009). Proteins were reduced with 20 mM dithiothreitol at 37°C for 30 min, followed by alkylation with 50 mM iodoacetamide at 37°C for 5 min in the dark using UA buffer (100 mM Tris/HCl, 8 M urea, pH 8.5) (Sigma-Aldrich, St. Louis, MO, United States). After three washes with UB buffer (100 mM Tris/HCl, 8 M urea, pH 8), proteins were digested with 0.3 µg LysC (Promega, Madison, WI, United States) in UC buffer (25 mM Tris/HCl, 2 M urea, pH 8) for 16 h at 37°C, followed by digestion with 0.15 µg trypsin in 50 mM ammonium bicarbonate (Promega) for 4 h at 37°C (Saveliev et al., 2013). Peptides were eluted, acidified with 0.1% formic acid, and desalted using STAGE extraction with self-packed Empore C18 tips (Merck, Sigma-Aldrich) (Rappsilber et al., 2003). The eluted peptides were concentrated, dissolved in 20 µL of 0.1% formic acid, and quantified using a Nanodrop 2000 (Thermo Fisher Scientific). A total of 1 µg of each sample was loaded onto a Vanquish Neo UHPLC nanoLC system coupled to an Exploris 480 mass spectrometer, using a 25 cm × 75 µm Acclaim PEPMap C18 column (Thermo Fisher Scientific). Peptides were separated using a 132-min binary gradient of water and acetonitrile, each containing 0.1% formic acid. Data-independent acquisition (DIA) was performed with an MS1 full scan (400–1200 m/z) at 120,000 resolution, an automatic gain control (AGC) of 3 × 10^6^, and a 50 ms maximum injection time, followed by 60 DIA windows (1 m/z overlap) optimized for placement. MS2 scans used a 30,000 resolution, AGC of 8 × 10^5^, and an automatic injection time. Fragmentation employed 30% normalized HCD energy. Data analysis was performed with DIA-NN (v1.8.1) (Demichev et al., 2020) with a predicted library generated from an *in silico* digested human UniProt reference database (UP000005640_9606), downloaded on 24.07.2023, allowing for K*/R* cuts, two missed cleavages, and a minimum peptide length of six. Statistical significance of alterations in protein abundance was assessed via a two-tailed Student’s t-test, with a false discovery rate (FDR)-adjusted p-value of 0.05 as the threshold for significance. Shared and exclusive proteins between Brew-EV and secretome, as well as DMEM-EV and secretome samples, were identified through Venn diagram analysis based on the presence/absence of protein IDs. The resulting protein subsets were subjected to gene ontology biological process (GOBP) enrichment analysis using the search tool for the retrieval of interacting genes/proteins (STRING) database (version 12.0) (Szklarczyk et al., 2023).

### 2.10 MiRNA expression profile

To extract total RNA from Brew- and DMEM-EVs (three samples per group), the pellet particles obtained from ultracentrifugation of the secretome were resuspended in 100 µL of PBS and transferred to Eppendorf tubes. Next, 700 µL of Qiazol (Qiagen, Germantown, MD, United States) was added, and RNA purification was performed using the miRNeasy Mini Kit (Qiagen). Reverse transcription was carried out using TaqMan UNIVERSAL MMix II (Applied Biosystems, Waltham, MA, United States), with either random priming or a miRNA-specific assay. Semi-quantitative PCR was performed using TaqMan-validated assays (Applied Biosystems). U6 (#001973) was used as the endogenous reference gene for cDNA in miRNA analysis. All experiments were performed in triplicate. Real-time data were collected using Microsoft Excel and analyzed with the 2^−ΔΔCT^ method for expression level determination, as previously described (Chinnici et al., 2021).

### 2.11 Activation of LX-2 cells and EV-based treatment

LX-2 cells (SCC064; Merck Millipore) from passages 2 to 6 were seeded onto treated 6-well tissue culture plates (Corning) at ∼250,000 cells per well in DMEM supplemented with 2% FBS. After 24 h, the cells were serum-starved overnight, and then treated with one of the following conditions: 1) vehicle control (PBS); 2) 10 ng/mL recombinant human TGF-β1 (Miltenyi); 3) 10 ng/mL TGF-β1 plus 0.17 mmol/l L-ascorbic acid (L-AA) 2-phosphate sesquimagnesium salt hydrate (Sigma-Aldrich). AD-MSC-EVs were tested at doses of 50,000 EVs per cell. Additionally, 1 mmol/L DMOG (Sigma-Aldrich) was used as a positive anti-fibrotic control (Smith-Cortinez et al., 2021). Both MSC-EVs and DMOG treatments were administered simultaneously with either TGF-β1 or TGF-β1/L-AA, as previously described (Smith-Cortinez et al., 2021). EVs were given in two doses (on days 1 and 2), while DMOG was administered as a single dose. Cellular and secreted protein samples were collected 48 h after the first dose of treatment. Given the detection of collagen in AD-MSC-derived EVs themselves (Supplementary Figure S1), potential collagen contamination contributing to observe effect was also evaluated. Non-activated LX-2 cells were treated with 50,000 EVs per cell, and Western blot analysis was performed on both cellular extracts and secreted fractions.

### 2.12 Extraction of cellular and secreted proteins and Western blot analysis

Cellular proteins from LX-2 cells were extracted using RIPA buffer, as described above. For secreted proteins, 1 mL of secretome was centrifuged at 2,000 × g for 10 min at 4°C to remove cell debris. The supernatant was subjected to trichloroacetic acid (TCA) precipitation (Sigma-Aldrich) overnight at 4°C, followed by centrifugation at 14,000 × g for 20 min at 4°C. The resulting protein pellet was reduced in 2% Laemmli sample buffer containing β-mercaptoethanol, with pH adjustment using 1 N NaOH. Twenty µg of cellular protein per lane were separated on 4%–15% gradient SDS-PAGE gels and transferred to PVDF membranes as described in the “Western blot analysis” subsection. Membranes were blocked and then incubated with primary antibodies against COL1A1 and α-SMA (Invitrogen), while β-actin was used as a loading control. Membrane imaging was performed as previously outlined. For densitometric analysis, band intensities were quantified using Image Lab software (version 6.1; Bio-Rad), and protein expression levels were normalized to those of non-activated LX-2 cells. Cell culture images were acquired using an inverted microscope (Olympus CKX41, Tokyo, Japan) equipped with a digital camera (Olympus U-TV0.5XC-3).

### 2.13 Statistics

Data were analyzed using GraphPad Prism version 8 (GraphPad Software, San Diego, CA, United States). For densitometric analysis (n = 4) and Luminex assays (n = 3), statistical significance was determined using unpaired t-tests, with each variable analyzed individually and without assuming equal SD. In the Luminex assay, which involved the simultaneous quantification of 20 cytokines, the Holm-Šidák correction was applied to account for multiple comparisons. Statistical analyses related to EV and secretome proteomics, as well as miRNA expression profiling, are described in the corresponding subsections. Results are presented as mean ± SD or median with interquartile range (IQR), as appropriate. A *p-*value of <0.05 was considered statistically significant. All experiments were conducted in at least triplicate, and statistical analyses were based on pooled data from a minimum of three independent experiments.

## 3 Results

### 3.1 Cell identity: immunophenotype analysis of AD-MSCs

**FIGURE 1:**
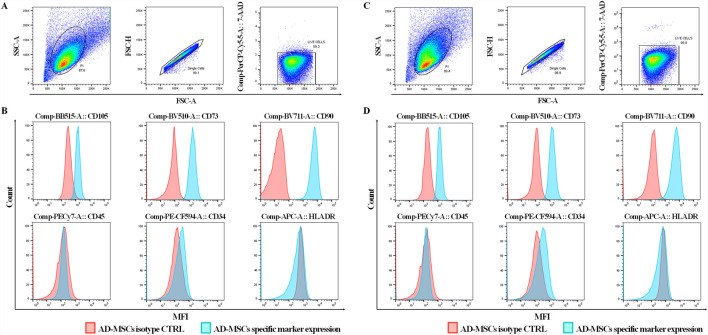
Immunophenotype analysis of AD-MSCs at P2 cultured in Brew and DMEM with 10% FBS. **(A)** Gating strategy of a Brew sample showing: 1) dot plots based on cell physical properties using FSC and SSC parameters; 2) dot plots to exclude doublets based on the correlation between FSC-A and FSC-H parameters; 3) dot plots excluding non-viable cells using the 7-AAD viability marker. **(B)** Representative flow cytometry histograms of AD-MSCs cultured in Brew medium, showing expression of MSC-positive markers (CD105, CD73, CD90) and negative markers (CD45, CD34, HLA-DR). **(C)** Dot plots of the DMEM sample showing the same gating strategies. **(D)** Corresponding histograms of AD-MSCs cultured in DMEM with 10% FBS, displaying the same set of markers. Red represents the isotype control, and blue indicates staining with the primary antibody. Abbreviations: P2, passage 2; FSC, forward scatter; SSC, side scatter; FSC-A, forward scatter area; FSC-H, forward scatter height; 7-AAD, 7-aminoactinomycin D.

Flow cytometry analysis of AD-MSCs cultured in either MSC-Brew GMP medium (Figures 1A,B) or DMEM with 10% FBS (Figures 1C,D) revealed consistent expression of surface antigens. All AD-MSCs were positive for classical MSC markers CD90, CD105, and CD73, and negative for hematopoietic markers CD34, CD45, and HLA-DR, regardless of culture medium or passage number. The results shown are from AD-MSCs at passage 2.

### 3.2 Purity/identity of AD-MSC-EVs: NTA and AFM analysis

**FIGURE 2:**
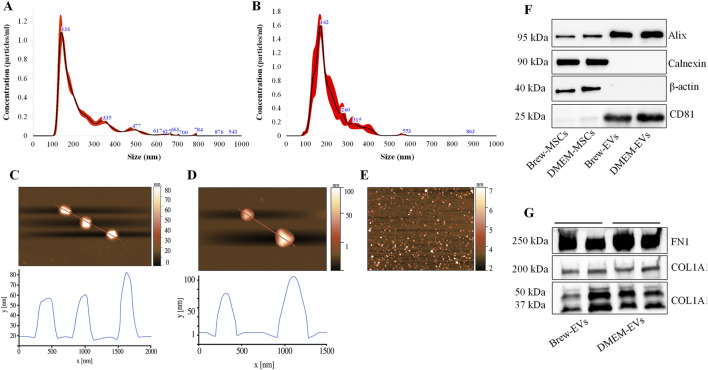
Purity and identity of AD-MSC-EVs by NTA, AFM and Western blot analysis. **(A)** Representative NTA histograms showing the size distribution and concentration of EVs isolated from the secretome of AD-MSCs cultured in Brew medium. **(B)** Representative NTA histograms showing the size distribution and concentration of EVs derived from the secretome of AD-MSCs cultured in DMEM with 10% FBS. **(C)** AFM image of Brew-EVs and the corresponded height profile. **(D)** AFM profile and height profile of DMEM-EVs. **(E)** AFM image of the PBS control. Brew-EVs and DMEM-EVs were analyzed at concentrations of 5 × 10^7^ and 7 × 10^7^ particles/mL, respectively. **(F)** Western blot analysis of total protein extracted from two representative AD-MSC samples cultured in Brew medium (lane 1) or DMEM with 10% FBS (lane 2), alongside their corresponding EVs (lanes 3, 4). The blot detects positive EV markers (Alix and CD81) and negative markers (calnexin and β-actin). **(G)** Evaluation of ECM protein content in Brew-EVs and DMEM-EVs, highlighting the presence of FN1 and cleavage fragments of COL1A1. Two representative samples for each type are shown. Abbreviations: NTA, Nanoparticle Tracking Analysis; AFM, atomic force microscopy; FBS, fetal bovine serum; ECM, extracellular matrix; FN1, fibronectin 1; COL1A1, collagen type I α1 subunit.

Particle size distribution histograms indicated a homogeneous particle population with minimal contamination from larger particles. Specifically, the average size of Brew-EVs was 125 ± 80 nm (n = 6) (Figure 2A), while DMEM-EVs (n = 6) exhibited an average size of 115 ± 70 nm (Figure 2B), both fitting the MISEV definition of small EVs (<200 nm). The EV concentration, obtained from 28 mL of secretome, was 3.4 ± 1.0 × 10^10^ particles/mL for Brew-EVs and 3.16 ± 0.75 × 10^10^ particles/mL for DMEM-EVs. AFM confirmed the presence of isolated, spherical or dome-shaped particles distributed across the mica surface for both Brew- and DMEM-EVs (Figures 2C,D). Particle heights were lower than the corresponding diameters measured by NTA, and ranged from 60 to 100 nm. Notably, no significant differences in morphology, size distribution, or surface cleanliness were observed between the Brew- and DMEM-EV preparations. No detectable particles were observed in the PBS control (Figure 2E).

### 3.3 Purity of AD-MSC-EVs: total protein content

The protein concentration of AD-MSC-EV batches was consistent with established ranges for MSC-EVs derived from adherent cultures and isolated via differential ultracentrifugation of serum-free secretome (Rohde et al., 2019). The particles-to-protein ratio (P/µg) (Webber and Clayton, 2013) for Brew-EVs was calculated using an average NTA value of 3.4 ± 0.10 × 10^10^ particles/mL and a corresponding protein concentration of 235.8 ± 35.5 μg/mL, resulting in a P/µg ratio of 1.45 × 10^8^. In comparison, DMEM-EVs, which exhibited a similar particle concentration (3.16 ± 0.07 × 10^10^) but significantly higher protein levels (433.3 ± 78 μg/mL; ***p < 0.001), displayed a lower P/µg ratio of 7.3 × 10^7^.

### 3.4 Identity of AD-MSC-EVs: positive and negative marker expression

Both Brew-EVs and DMEM-EVs tested positive for Alix and CD81, while showing no expression of Calnexin and β-actin, in contrast to the opposite pattern observed in their parent cells (Figure 2F). Additionally, both EV types were enriched with ECM proteins, including FN1 and collagen (Figure 2G). Collagen expression in AD-MSC-EVs exhibited prominent bands at ∼50 and 37 kDa, which likely correspond to collagen cleavage products, whereas procollagen bands at 200 and 180 kDa were only weakly detected (Figure 2G). The full Western blot membranes are shown in Supplementary Material S1. Notably, collagen enrichment in AD-MSC-EVs was significantly higher compared to non-dermal MSC-EVs, such as those derived from umbilical cord (UC)-MSC-EVs (Supplementary Figure S1).

### 3.5 Identity of AD-MSC-EVs: low inflammatory cytokine content

**FIGURE 3:**
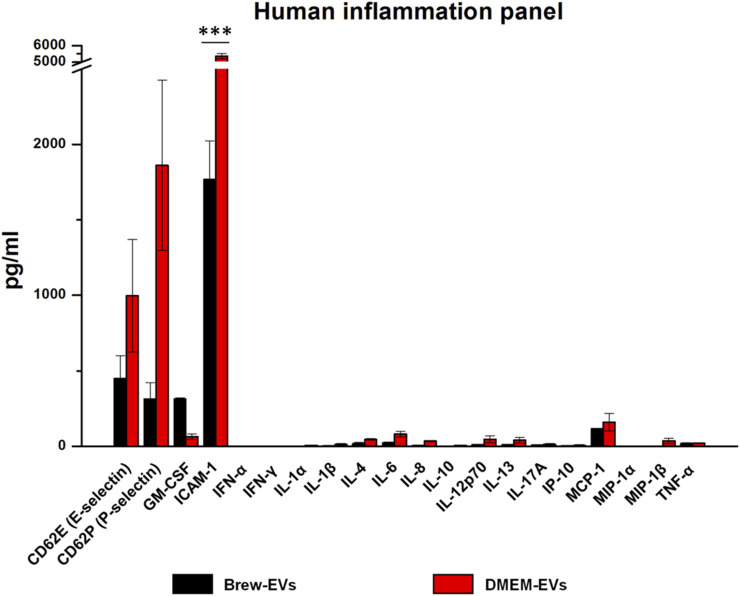
Identity of AD-MSC-EVs: inflammatory cytokine profiling. Luminex analysis of 20 cytokines from the Human Inflammation Panel, including selectins and ICAM-1, performed on EVs isolated from AD-MSCs cultured in Brew medium or DMEM with 10% FBS. Cytokine levels are expressed in pg/mL. Statistical differences were assessed using multiple unpaired t-tests with Holm-Šidák correction for multiple comparisons (***p < 0.001). Each cytokine was analyzed independently without assuming a uniform standard deviation (SD).

Luminex-based analysis revealed a near absence of pro-inflammatory cytokines in EVs derived from both Brew and DMEM culture conditions. Adhesion molecules and selectins were present in both EV types, with higher concentrations detected in DMEM-EVs. Specifically, Brew-EVs showed lower levels of E-selectin (450 ± 150 pg/mL vs. 997 ± 373 pg/mL), P-selectin (313 ± 109 pg/mL vs. 1861 ± 564 pg/mL), and ICAM-1 (1768 ± 253 pg/mL vs. 5371 ± 165 pg/mL) compared to DMEM-EVs. Among these, only ICAM-1 showed a statistically significant difference (***p < 0.001), likely due to the high standard deviation observed in the DMEM-EV group, which may have masked significance in the other markers. In contrast, granulocyte-macrophage colony-stimulating factor (GM-CSF) was more abundant in Brew-EVs than in DMEM-EVs (313 ± 7.6 pg/mL vs. 64.6 ± 18.5 pg/mL), although this difference was not statistically significant (Figure 3).

### 3.6 Identity of AD-MSC-EVs: proteomic profile

**FIGURE 4:**
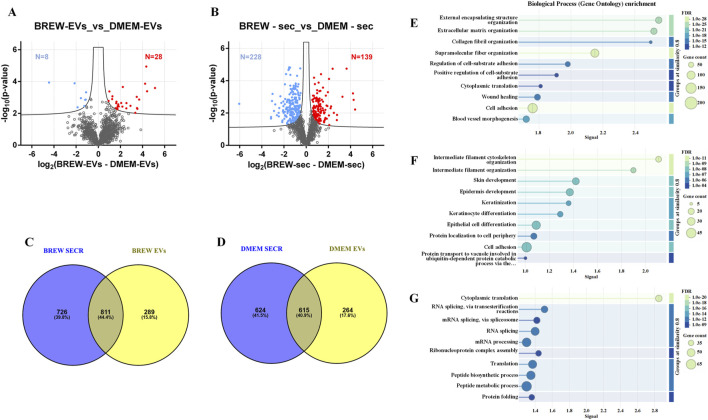
LC-MS/MS proteomic analysis of EVs and secretome from AD-MSCs cultured in Brew medium or DMEM with 10% FBS. **(A)** Volcano plot showing differentially expressed proteins between Brew- and DMEM-derived EVs. Upregulated proteins in Brew-EVs are shown in red, while those in DMEM-EVs are shown in blue. **(B)** Volcano plot of differentially expressed proteins in Brew- and DMEM-derived secretomes. Red indicates upregulated proteins in Brew-secretome, and blue indicates upregulated proteins in DMEM-secretome. The x-axis represents log_2_ fold change (Brew/DMEM), and the y-axis shows the -log_10_ *p-*value. Statistically significant proteins are indicated above the FDR threshold (black dashed lines). **(C)** Venn diagrams illustrating shared and exclusive proteins in Brew-derived secretome and EVs. **(D)** Venn diagrams illustrating shared and exclusive proteins in DMEM-derived secretome and EVs. **(E)** GOBP enrichment analysis of proteins shared between Brew-derived EVs and secretome. **(F)** GOBP enrichment of proteins exclusive to Brew-EVs. **(G)** GOBP enrichment of proteins exclusive to the Brew-secretome. Analyses were performed using STRING. Abbreviations: FDR, false discovery rate; secr, secretome; GOBP, gene ontology biological process; STRING, search tool for the retrieval of interacting genes/proteins.

Mass spectrometry analysis of AD-MSC-derived EVs identified 1,258 proteins, with 28 significantly more abundant in Brew-EVs and 8 enriched in DMEM-EVs (Figure 4A; Supplementary Material S2). In comparison, the secretome contained 1,910 proteins, including 139 enriched in the Brew condition and 228 in the DMEM condition (Figure 4B; Supplementary Material S3). Overall, the secretome displayed a broader protein repertoire than the EV fractions, as shown by the comparative Venn diagrams (Figures 4C,D; Supplementary Material S4). STRING analysis indicated that while both Brew and DMEM samples shared general biological process enrichment, specific patterns emerged in the Brew condition. In particular, proteins shared between Brew-EVs and the Brew-secretome were enriched in processes related to *“wound healing”* and *“blood vessel morphogenesis”* (Figure 4E), suggesting pro-regenerative properties. Proteins exclusive to the EV fraction, whether Brew- or DMEM-derived, were significantly associated with pathways involved in *“skin development”* and *“epidermis development”,* likely reflecting the dermal origin of the donor MSCs (Figure 4F; Supplementary Figure S2). Conversely, proteins unique to the secretome, whether Brew- or DMEM-derived, did not show significant enrichment in biological processes directly relevant to the focus of the study (Figure 4G; Supplementary Figure S2).

### 3.7 Identity of AD-MSC-EVs with respect to target disease: anti-fibrotic miRNA content

**FIGURE 5:**
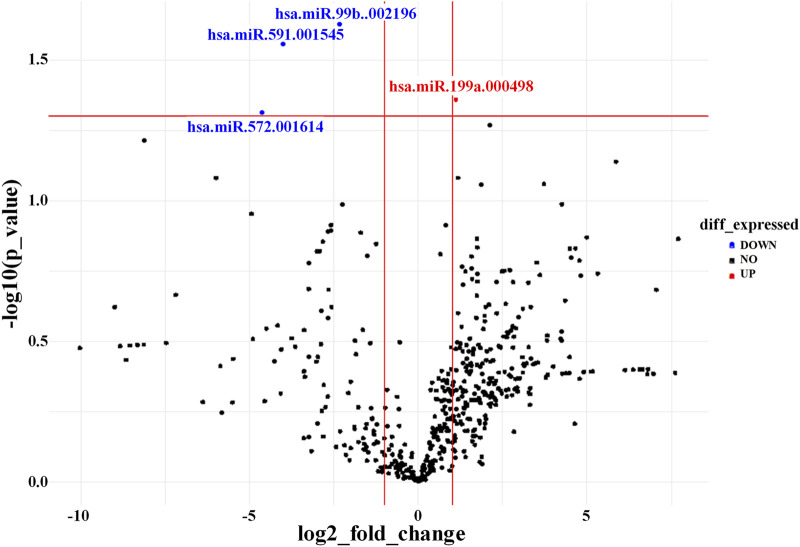
AD-MSC-EV identity based on miRNA expression profile. Volcano plot showing differentially expressed miRNAs between Brew-EVs and DMEM-EVs. The plot displays the log2 fold change in miRNA expression versus the -log10 p*-*value. MiRNAs significantly upregulated in Brew-EVs are highlighted in red, while those significantly upregulated in DMEM-EVs are shown in blue.

The miRNA expression profiles of EVs derived from AD-MSCs cultured in Brew or DMEM medium were largely similar, with only four miRNAs showing differential expression out of ∼750 analyzed (Figure 5). Specifically, miR-199a-5p was upregulated in Brew-EVs, while miR-99b-3p, miR-591, and miR-572 were downregulated. Both Brew- and DMEM-EVs exhibited comparable profiles of anti-fibrotic miRNAs, with similar expression patterns and intensities across the two culture conditions (Table; Supplementary Material S5). Notably, target prediction analysis using DIANA-miRPath (Tastsoglou et al., 2023) revealed that miR-199a-5p targets seven genes (*LAMB2, THBS1, COL6A1, COL5A1, COL1A1, FN1*, and *CD44*) within the signaling pathway “*ECM-receptor interaction”,* as defined by the Kyoto Encyclopedia of Genes and Genomes (KEGG) (Supplementary Material S5).

### 3.8 Biological activity of AD-MSC-EVs: inhibition of collagen secretion and α-SMA expression in LX-2 cells

**FIGURE 6:**
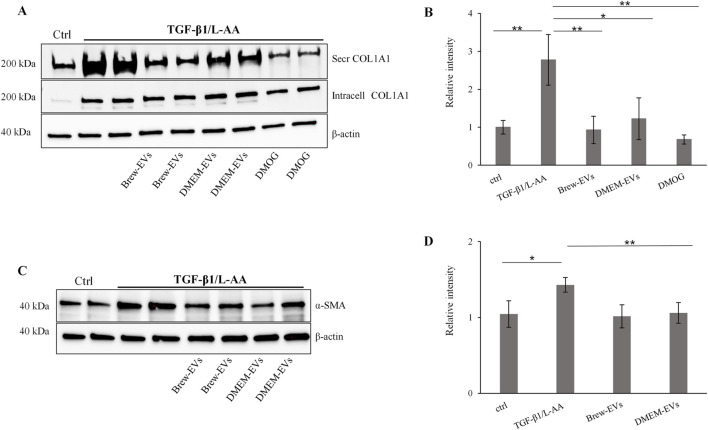
*In vitro* biological activity of AD-MSC-EVs in a liver fibrosis-relevant model. **(A)** Representative Western blot showing secreted and intracellular COL1A1 expression in non-activated LX-2 cells (lane 1, control) and in LX-2 cells activated with TGF-β1 and L-AA for 48 h (lanes 2–9). AD-MSC-EVs were administered at 50,000 EVs per cell on days 1 and 2. Brew-EV treatment is shown in lanes 4-5, DMEM-EV treatment in lanes 6-7, and positive control DMOG treatment in lanes 8–9. β-actin was used as a loading control. **(B)** Densitometric analysis of secreted COL1A1 in LX-2 cells. **(C)** Representative Western blot showing α-SMA levels in non-activated LX-2 cells (lane 1, control) and in LX-2 cells activated with TGF-β1 and L-AA for 48 h (lanes 2–8). AD-MSC-EVs were administered at 50,000 EVs per cell on days 1 and 2. Brew-EV treatment is shown in lanes 5-6, and DMEM-EV treatment in lanes 7–8. **(D)** Densitometric analysis of α-SMA in LX-2 cells. Statistical significance was assessed using unpaired t-tests to compare non-activated versus activated groups, and activated untreated versus activated EV- or DMOG-treated groups (Figure 6B: *p < 0.05; **p < 0.01; ***p < 0.001); (Figure 6D: *p < 0.05; **p < 0.01). Abbreviations: ctrl, control; L-AA, ascorbic acid; secr, secreted; intr, intracellular; DMOG, dimethyloxalylglycine.

**FIGURE 7:**
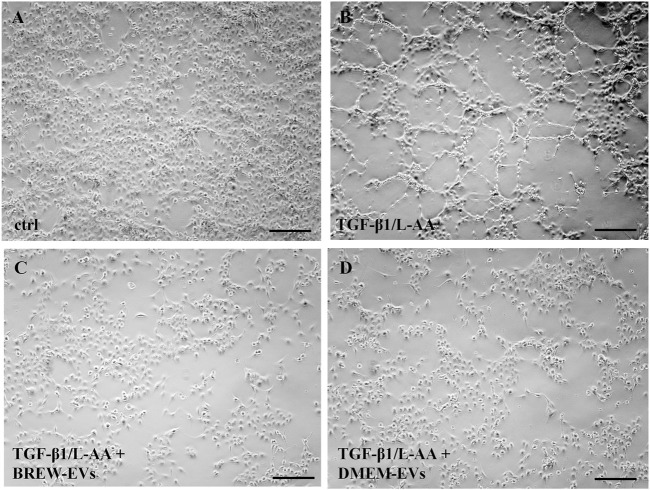
Morphological changes in LX-2 cells upon activation and EV treatment. **(A)** Control, non-activated LX-2 cells. **(B)** LX-2 cells displaying a network-like morphology on day 3 following activation with TGF-β1 and L-AA. **(C)** LX-2 cells co-treated with TGF-β1/L-AA, and two doses of 50,000 Brew-EVs per cell. **(D)** LX-2 cells co-treated with TGF-β1/L-AA, and two doses of 50,000 DMEM-EVs per cell. Abbreviations: L-AA, L-ascorbic acid; Scale bar: 100 µm.

The anti-fibrotic activity of AD-MSC-EVs was assessed by evaluating their ability to inhibit collagen levels in TGF-β1-activated LX-2 cells, with or without co-stimulation by L-AA. Treatment with AD-MSC-EVs significantly reduced secreted collagen; however, no detectable effect was observed on intracellular collagen expression (Figure 6A). A single dose of 50,000 EVs per cell did not reduce collagen secretion (data not shown), while two sequential doses at the same concentration were required to achieve effective inhibition. In contrast, a single dose of DMOG was sufficient to suppress collagen secretion (Figures 6A,B). EV treatment also reduced α-SMA expression (Figures 6C,D), with the effect more evident in cells co-activated with TGF-β1 and L-AA compared to TGF-β1 alone (Supplementary Figure S3A,B). Both Brew- and DMEM-EVs exhibited comparable anti-fibrotic activity. Full Western blot membranes are provided in Supplementary Material S6. Morphologically, TGF-β1/L-AA activation induced a network-like pattern in LX-2 cells, which was absent in untreated controls (Figures 7A,B). Treatment with EVs from either Brew or DMEM cultures markedly reduced this network formation (Figures 7C,D).

Regarding potential EV-associated protein contamination, no significant differences in intracellular collagen levels were observed between untreated and EV-treated LX-2 cells (Supplementary Figure S4). However, faint collagen bands (∼50 and ∼35 kDa) were detected in the secretome of EV-treated LX-2 cells (Supplementary Figure S5).

## 4 Discussion

The use of xeno-free, GMP-compliant culture media is a critical step toward advancing both cell-based and EV-based therapeutics. Such media not only enhance safety and regulatory compliance but also promote the clinical translation of regenerative therapies. In line with the MISEV guidelines, our study integrates well-defined, ethically sourced cell types, GMP-compliant and serum-free culture conditions, standardized EV isolation methods, and rigorous functional characterization. We demonstrate the feasibility of isolating MSCs from human dermal tissue (Chinnici et al., 2014; 2021; Tastsoglou et al., 2023), specifically surgical skin remnants from healthy adult donors (Iannolo et al., 2024). This approach provides an ethically acceptable and abundant cell source from biological material that would otherwise be discarded. Using MSC-Brew GMP Medium offered a robust platform for the isolation and expansion of AD-MSCs under GMP-like conditions. Cells cultured in this medium maintained a stable immunophenotype, indicating that it effectively preserves essential cellular properties.

In addition to the stable AD-MSC immunophenotype, we observed minor differences in EV characteristics depending on the culture medium. Both Brew- and DMEM-EVs met the MISEV guidelines, adhering to established criteria for size, purity and identity, including the absence of calnexin, indicative of minimal cellular contamination (Webber and Clayton, 2013). Our AD-MSC-derived EV preparations were predominantly composed of “small” EVs, consistent with the MISEV definition of EVs. To ensure accurate characterization, we combined NTA and AFM as complementary techniques, following MISEV2023 guidelines that recommend using at least two orthogonal methods to distinguish EVs from contaminants (Saint-Pol and Culot, 2025; Welsh et al., 2024). This approach enhances the reliability of EV characterization and supports the purity and consistency of our preparations, which is critical for their clinical translation. As expected, EV heights measured by AFM were smaller than diameters measured by NTA. This is because EVs flatten on the mica surface during AFM imaging, reducing their height, while NTA measures the hydrodynamic diameter in suspension, which includes the hydration layer and surface proteins, resulting in larger size estimates. (Vorselen et al., 2020). Notably, EVs derived from AD-MSCs cultured in Brew medium exhibited a significantly higher particle-to-protein ratio (Webber and Clayton, 2013) compared to those cultured in DMEM with 10% FBS, suggesting enhanced purity. This improved purity is a significant advantage for clinical applications, as it reduces potential contaminants and enhances the therapeutic potential of MSC-EVs. The increased purity is likely attributed to the absence of serum in Brew, minimizing protein contamination in the EV preparations. Despite a two-day starvation period and multiple washes prior to serum-free secretome collection, minor traces of serum proteins may still remain. As this is the first study to explore the impact of Brew medium on MSC-EVs, further research is needed to validate these findings and better understand how culture conditions influence EV purity.

Both EV types were subjected to Luminex-based identity testing, which confirmed negligible levels of inflammatory cytokines, consistent with established MSC-EV identity criteria (Rohde et al., 2019). Brew-EVs showed a trend toward higher GM-CSF levels compared to DMEM-EVs, although the difference was not statistically significant. GM-CSF has a dual role in immune activation and tissue regeneration, which may have biological implications depending on the therapeutic context. Nonetheless, since the measured concentrations remained below 0.5 ng/mL, these levels are unlikely to raise safety concerns. DMEM-EVs were enriched in adhesion molecules such as ICAM-1, E-selectin, and P-selectin, which may enhance their interaction with target cells (Bui et al., 2020; Singh et al., 2021; Noris and Galbusera, 2023). However, due to the high variability, these differences were not statistically significant. The greater variability observed in DMEM-EVs compared to Brew-EVs likely reflect the influence of culture medium. Non-GMP-grade media are prone to batch variability and uncontrolled fluctuations in nutrient composition, pH, and growth factors, potentially impacting EV profiles. In contrast, GMP-grade media like Brew offer a standardized environment that reduces such variability. We hypothesize that this consistency supports the production of more uniform EV populations.

Proteomic analysis revealed that EV composition was largely conserved across both Brew and DMEM culture conditions, with only ∼2.8% of EV proteins showing differential abundance. This limited variability could reflect the regulated nature of EV biogenesis, which is thought to involve conserved pathways that selectively package proteins, RNAs, and lipids (Lim et al., 2021; Teng and Fussenegger, 2021), potentially resulting in a more consistent molecular profile. In contrast, the two secretome types appear to exhibit greater variability, with ∼19% of proteins differentially abundant despite being collected under identical serum-free conditions. This increased heterogeneity may be attributed to the complex and dynamic nature of the secretome, which includes soluble proteins that may respond to subtle culture-dependent factors such as residual serum components, oxidative stress, or nutrient fluctuations (Mallia et al., 2020). Notably, in Brew samples, the proteins shared between EVs and the secretome were specifically enriched for GOBP terms “wound healing” and “blood vessel morphogenesis” (Figure 4D). These enrichments were not observed in the corresponding DMEM-derived profiles (Supplementary Material S5), suggesting that Brew-associated factors may possess a higher regenerative potential. Overall, these findings suggest that EV-based therapies may offer greater stability and reproducibility than secretome-based approaches. Additionally, Venn diagrams, showing fewer exclusive EV proteins compared to secretome exclusive proteins further support that EV biogenesis selectively packages a smaller, more specific protein set than the broader, more diverse secretome.

Given the well-established anti-fibrotic properties of MSC-derived EVs (Bruno et al., 2020), the anti-fibrotic miRNA profile could be incorporated as a disease-relevant “informative” test, as suggested (Rohde et al., 2019). This test could be applied to MSC-EV batches to standardize and enhance their therapeutic potential in the treatment of fibrotic diseases. Notably, both Brew- and DMEM-derived EVs in our study were similarly enriched in miRNAs previously identified as anti-fibrotic. Among the highly expressed anti-fibrotic miRNAs in AD-MSC-EVs (Table 1), we have previously identified miRNAs, such as miR-29a-3p, some members of the let-7 family (particularly miR-7a-5p and miR-7b-5p), and miR-143-3p, to be highly expressed in EVs derived from MSCs of other sources, such as umbilical cord and fetal liver (data not shown). Therefore, given their consistent expression across different MSC types, we propose that they could represent a potential molecular signature for MSC-derived EVs in anti-scarring therapies.

**TABLE 1:** Identity of AD-MSC-EVs: anti-fibrotic miRNA profile in Brew and DMEM-EVs.

miRNA name	Expression intensity in Brew-EVs	Expression intensity in DMEM-EVs	References
hsa-let-7a-5p	High	High	Zhang et al. (2019)
hsa-let-7b-5p	High	High	Sun et al. (2021)
hsa-miR-15b-3p	Aver	Aver	Ma et al. (2021)
hsa-miR-16-5p	High	High	Ma et al. (2021)
hsa-miR-19b-3p	High	High	Lakner et al. (2012)
hsa-miR-24-3p	High	High	Wan et al. (2022)
hsa-miR-25-3p	Aver	Aver	Genz et al. (2019)
hsa-miR-27b-3p	Aver	Aver	Wan et al. (2022)
hsa-miR-29a-3p	High	High	Xuan et al. (2017), Lin et al. (2019), Fu et al. (2020)
hsa-miR-29b-3p	Low	Low	Liang et al. (2016)
hsa-miR-30a-5p	High	High	Zheng et al. (2018)
hsa-miR-30c-5p	High	High	Gu et al. (2021)
hsa-miR-130a-3p	Aver	Aver	Wang et al. (2017), Liu et al. (2021)
hsa-miR-143-3p	High	High	Tu et al. (2020)
hsa-miR-145-5p	High	High	Zhou et al. (2016)
hsa-miR-146a-5p	Aver	Aver	Zou et al. (2017), 2019
hsa-miR-152-3p	High	High	Li et al. (2019)
hsa-miR-193b-3p	High	High	Ju et al. (2019)
hsa-miR-223-3p	Aver	Aver	He et al. (2019), Wang et al. (2021)
hsa-miR-370-3p	Aver	Aver	Lu et al. (2015)
hsa-miR-483-5p	Aver	Aver	Niture et al. (2023)
hsa-miR-494-3p	Aver	Aver	Li et al. (2021)

These results suggest that culture conditions did not significantly influence the anti-fibrotic potential of AD-MSC-EVs. While the miRNA profiles were largely comparable, with only four miRNAs showing differential expression, miR-199a-5p was upregulated in Brew-EVs. miR-199a-5p is predicted to target genes within the “*ECM-receptor interaction*” pathway, all of which are involved in fibrosis (Wang et al., 2020; Osawa et al., 2021; Zhang et al., 2024). Despite the controversial role of miR-199a-5p in liver diseases, with both protective (Dai et al., 2013) and detrimental (Lino Cardenas et al., 2013) effects, its upregulation in Brew-EVs may contribute to their anti-fibrotic activity.

To evaluate the biological activity of AD-MSC-EVs, we quantified the number of EV particles per target cell, as this metric offers greater consistency than protein concentration-based approaches, which are often affected by non-EV protein contamination (Pachler et al., 2017). We intentionally refer to these outcomes as indicative of EV “biological activity” rather than “potency,” since the latter term is generally reserved for rigorously validated, quantitative measures of therapeutic efficacy (Bravery et al., 2013). Although a full proteomic characterization of the AD-MSC-EVs was beyond the scope of this study, our analysis confirmed the presence of ECM-related proteins, consistent with previous reports (van Balkom et al., 2019). Notably, several collagen types were detected among the shared proteins between EVs and the secretome, rather than being unique to the EV fraction (Figures 4D,E; Supplementary Material S4). To exclude the possibility that EV-associated collagen contributed to the observed effects, we conducted Western blot analysis using an anti-COL1A1 antibody. This revealed two distinct bands (∼50 and ∼37 kDa) in AD-MSC-EVs, likely corresponding to C-terminal collagen fragments (Iannarone et al., 2019). These differed markedly from the ∼200 kDa procollagen band observed in LX-2 cells, suggesting that EV-associated collagen was unlikely to interfere with our assay and supporting the validity of our collagen quantification.

Notably, the combination of TGF-β1 with the prolyl hydroxylase cofactor L-AA to activate LX-2 cells significantly enhanced collagen secretion in LX-2 cells compared to TGF-β1 alone. L-AA also caused a slight but statistically significant upregulation of α-SMA expression compared to non-activated control cells, allowing quantifiable assessment of EV effects and enabling a more robust evaluation of the biological activity of AD-MSC-EVs. These findings align with previous reports demonstrating that L-AA enhances collagen secretion *in vitro* in both LX-2 cells and primary hepatic stellate cells (Smith-Cortinez et al., 2021), and further supported by findings that L-AA enhances TGF-β1-induced myofibroblast differentiation (Piersma et al., 2017). Furthermore, we found that both AD-MSC-EVs and the pan-hydroxylase inhibitor DMOG effectively inhibited TGF-β1/L-AA-induced collagen secretion in LX-2 cells, without affecting intracellular collagen levels. This suggests a common anti-fibrotic mechanism involving the inhibition of P4H enzymes (Smith-Cortinez et al., 2021). Additionally, AD-MSC-EVs contain anti-fibrotic miRNAs (Table 1), including miR-30a-5p which is predicted to target the *P4HA2* gene (Cui et al., 2025). This further suggests that miRNA-mediated inhibition of collagen production may contribute to the observed anti-fibrotic effects of AD-MSC-EVs. Given the potential role of P4Hs in fibrosis across various organs (Kant et al., 2013; Luo et al., 2015; Zhang et al., 2023), further studies are needed to elucidate the specific miRNA pathways involved in modulating collagen dynamics.

In conclusion, our findings demonstrate that MSC-Brew GMP Medium provides a robust and reproducible platform for deriving AD-MSCs and producing high-quality EVs for therapeutic use. This serum- and xeno-free culture medium not only preserves MSC properties but also enhances EV purity, making it a promising approach for treating fibrosis-related disorders. Importantly, this study addresses a critical gap by providing the first comprehensive evaluation of the impact of MSC-Brew GMP Medium on the functional characteristics of MSC-derived EVs. Further studies should focus on evaluating the therapeutic efficacy and long-term stability of these AD-MSC-EVs across various disease models to fully assess their clinical potential. This study marks a significant step toward standardizing MSC-EV production for clinical use and could accelerate the development of MSC-EV-based therapies in regenerative medicine.

## Data Availability

The datasets presented in this study can be found in online repositories. The names of the repository/repositories and accession number(s) can be found in the article/Supplementary Material.
