# Supplementary material for: GMP-compliant, serum-free cultures preserve therapeutic potential of extracellular vesicles from human mesenchymal stromal cells
Source: Front Cell Dev Biol. 2025 Sep 1;13:1633912. doi: 10.3389/fcell.2025.1633912 (PMC12434029; doi:10.3389/fcell.2025.1633912)
Supplement: Supplementary file 3 [file DataSheet1.pdf]

Supplementary material S1: full WB membranes of figure 2F and 2G.

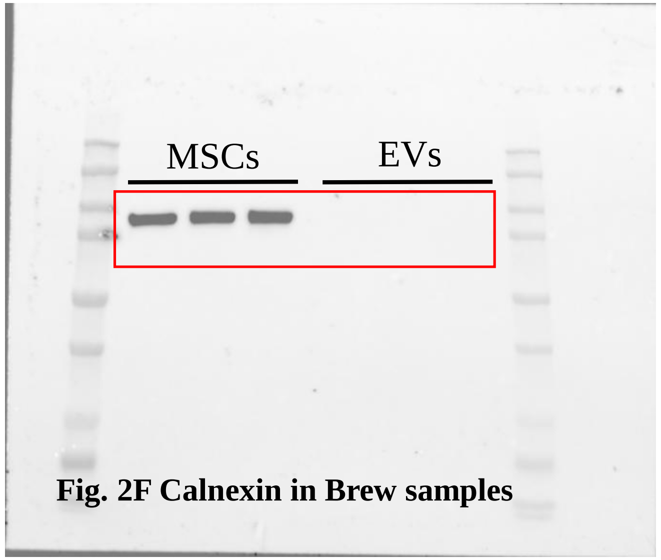

Fig. 2F Calnexin in Brew samples

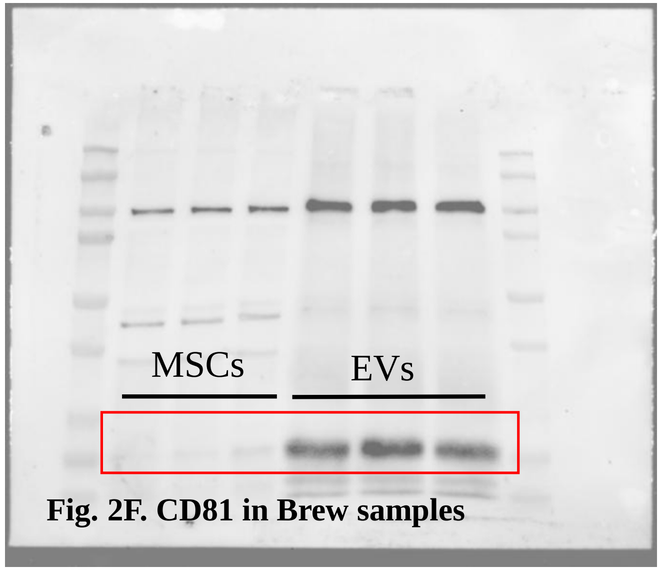

Fig. 2F. CD81 in Brew samples

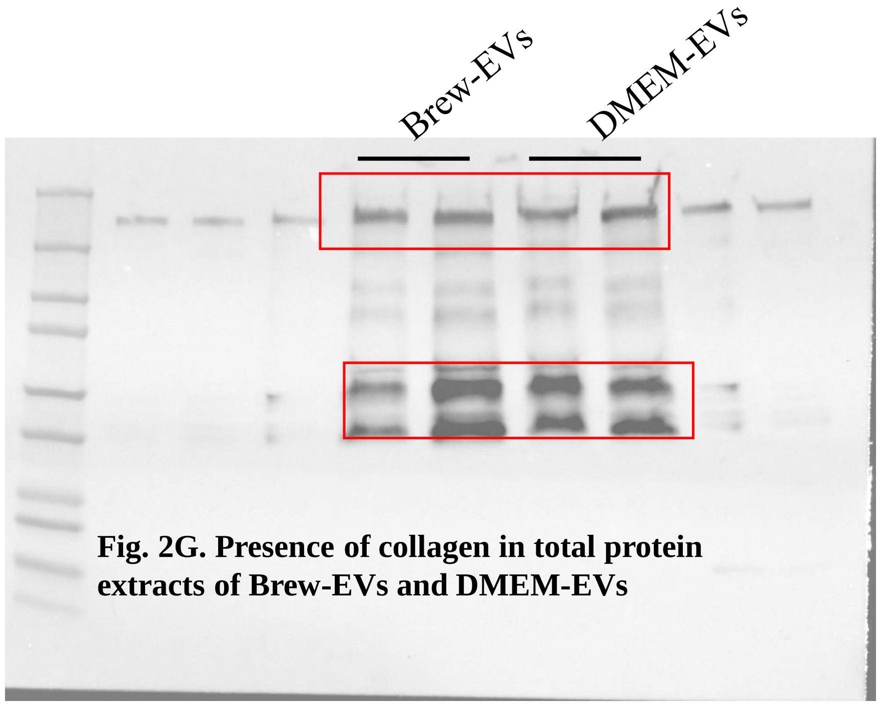

Fig. 2G. Presence of collagen in total protein extracts of Brew-EVs and DMEM-EVs

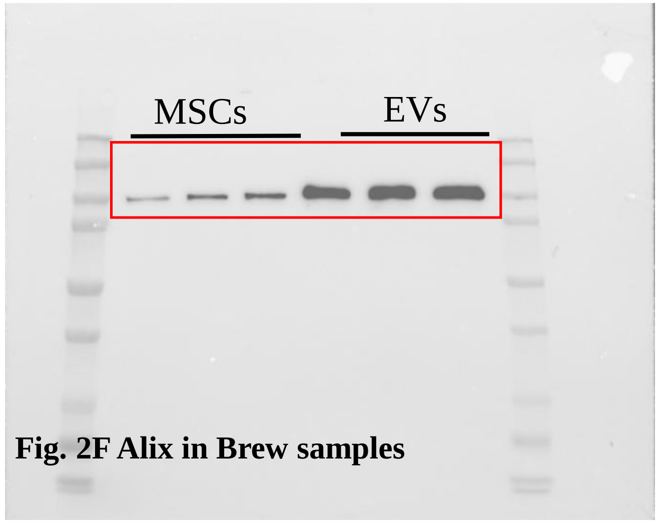

Fig. 2F Alix in Brew samples

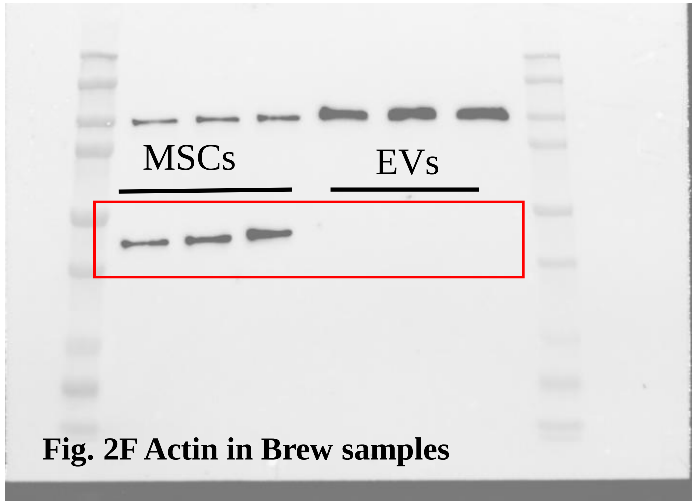

Fig. 2F Actin in Brew samples

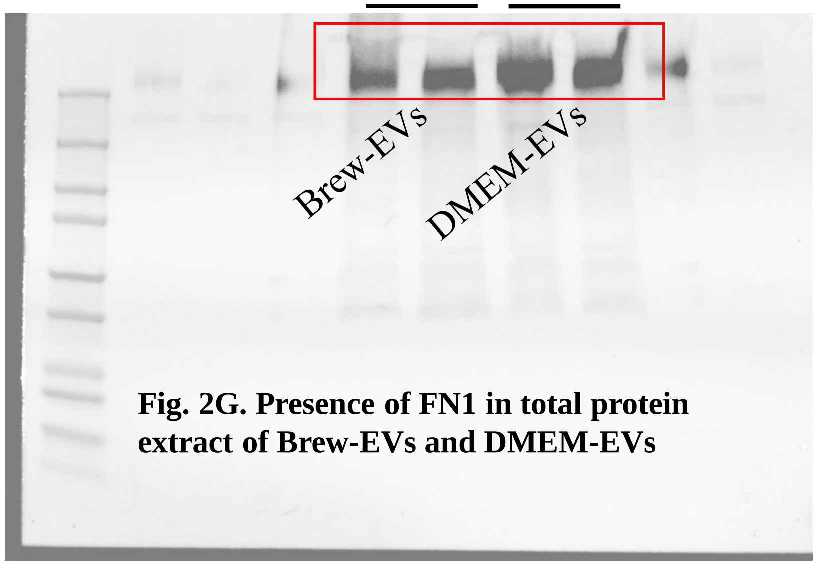

Fig. 2G. Presence of FN1 in total protein extract of Brew-EVs and DMEM-EVs

S1

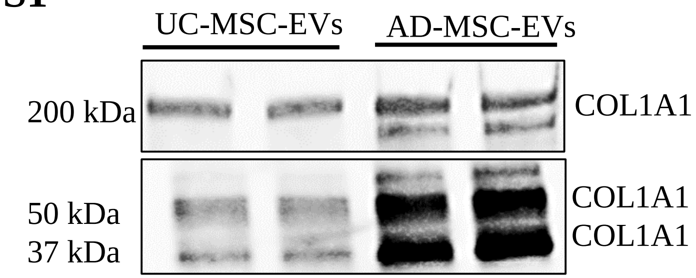

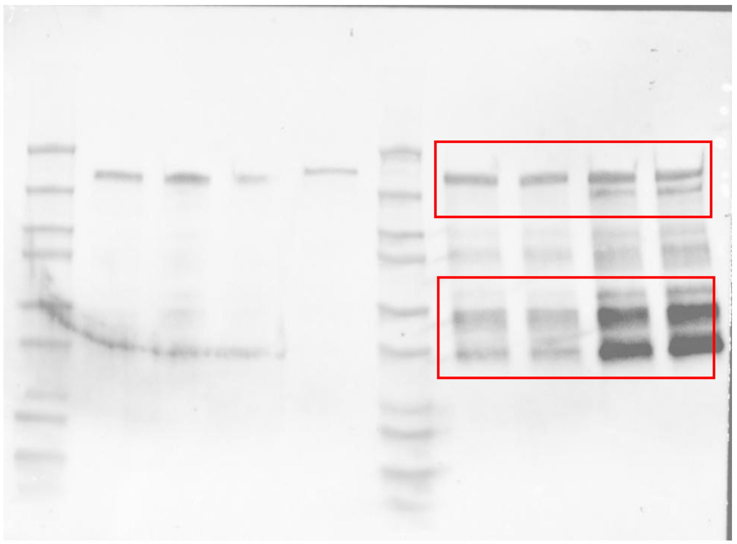

Supplementary figure S1. Western blot analysis of RIPA-extracted EV proteins using an anti-COL1A1 antibody. EV collagen content in RIPA-extracted proteins of EVs derived from UC-MSCs and AD-MSCs. AD-MSC-EVs show collagen enrichment compared to UC-MSC-EVs. Two representative samples for each EV type are shown. Abbreviations: UC, umbilical cord; AD, adult dermis.

Supplementary figure S2

Shared DMEM secretome/EV proteins

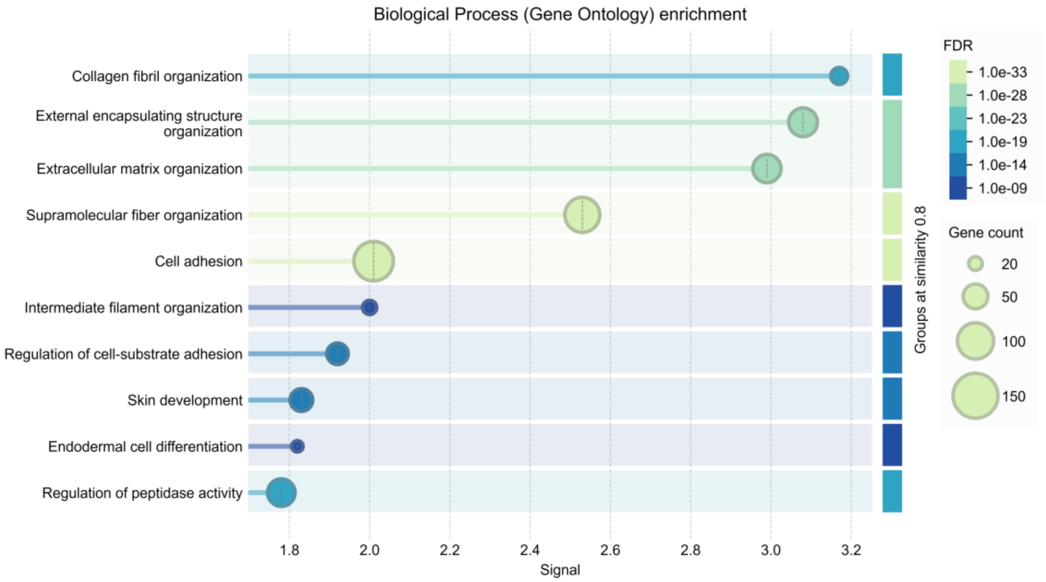

Exclusive DMEM EV proteins

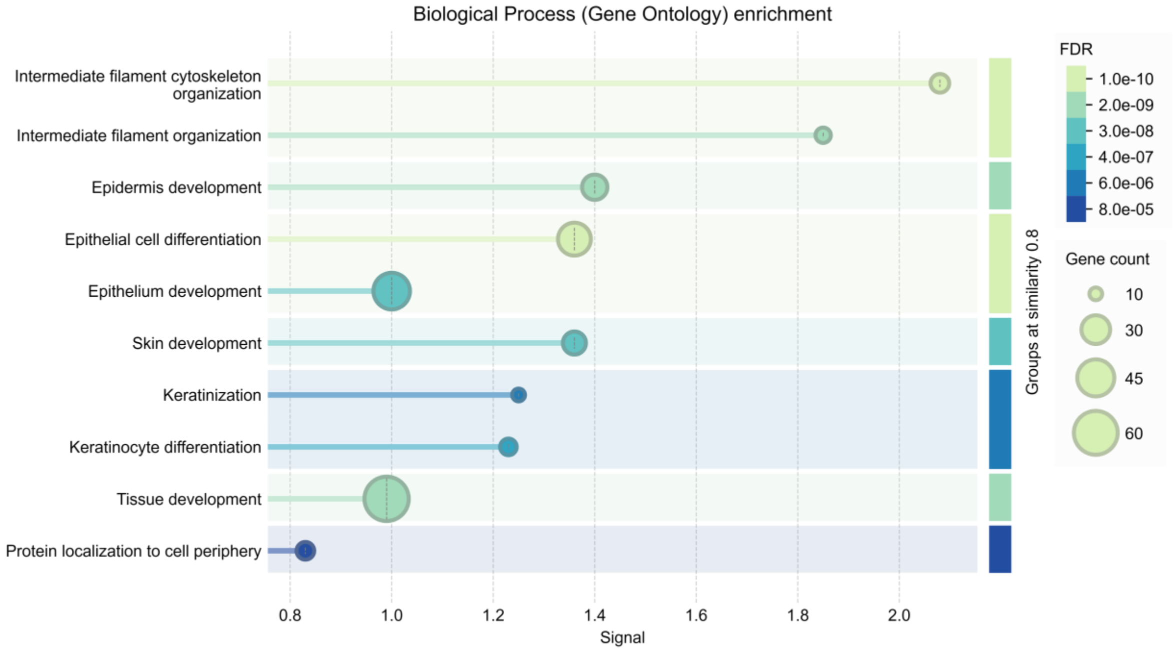

Exclusive DMEM secretome proteins

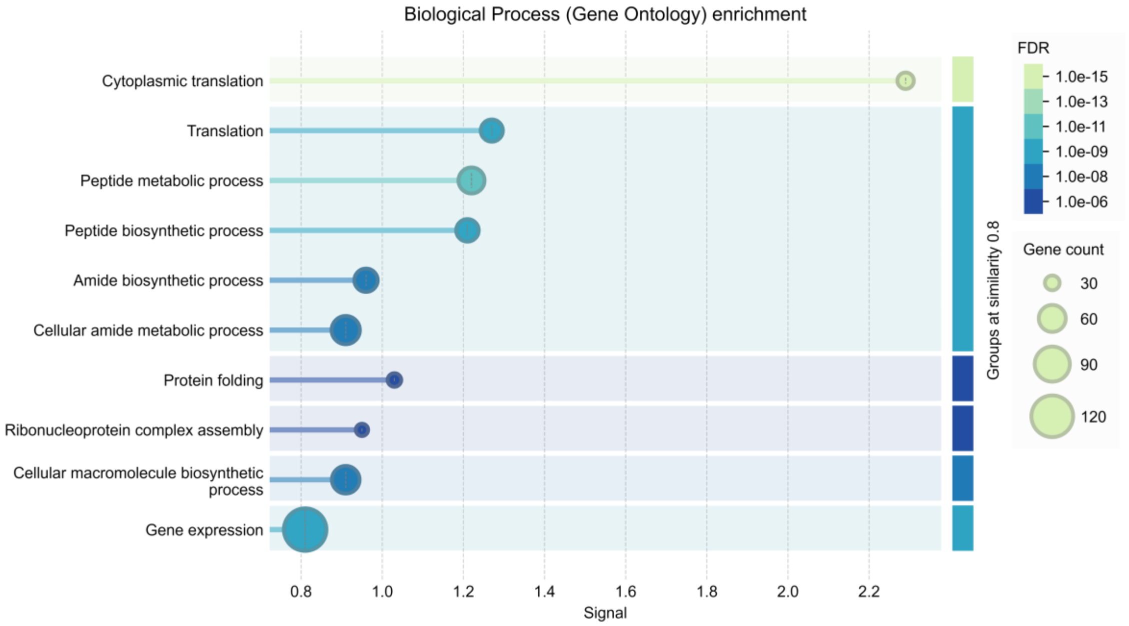

S3A

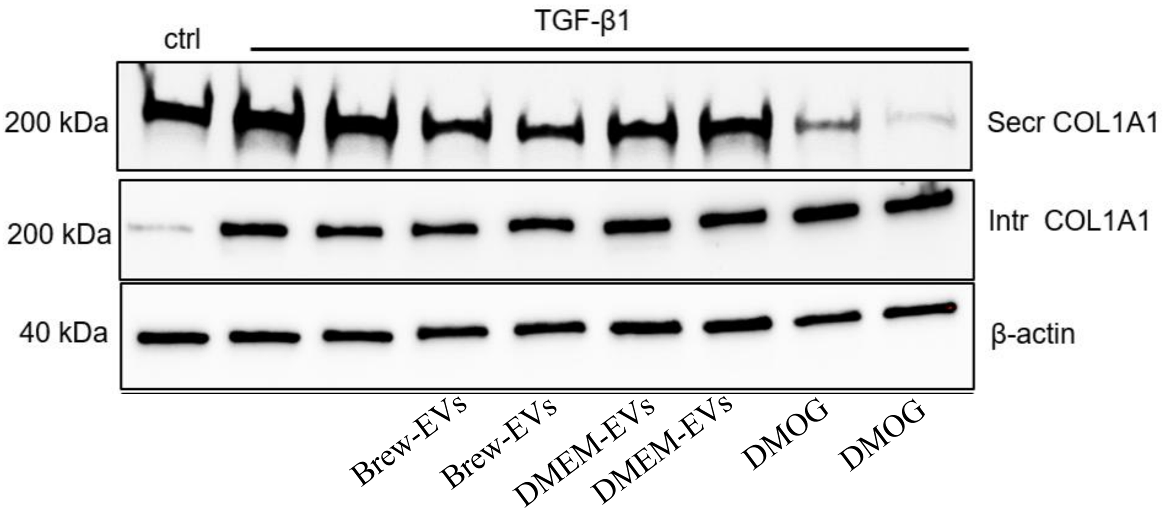

S3B

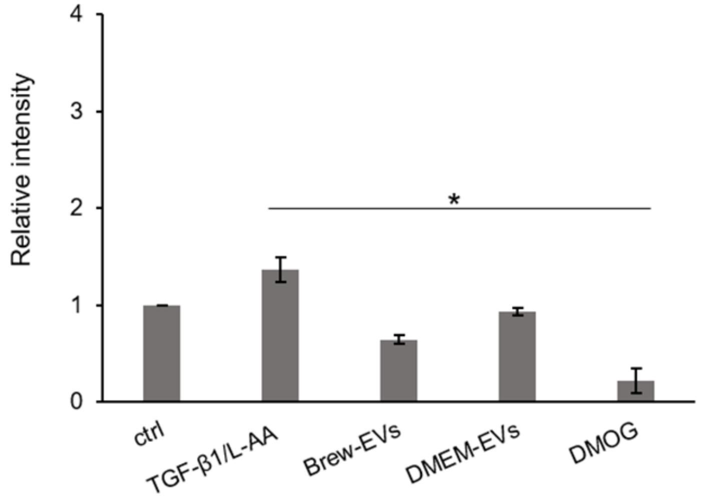

Supplementary figures S3. (A) Representative Western blot of secreted and intracellular COL1A1 expression in LX-2 cells activated with TGF-β1 only, with treatments as in panel A. β-actin was used as a loading control. (B) Densitometric analysis of secreted COL1A1 in LX-2 cells activated with TGF-β1 only. Statistical significance was assessed using unpaired t-tests to compare non-activated versus activated groups, and activated untreated versus activated EV- or DMOG-treated groups (*$p<0.05$).

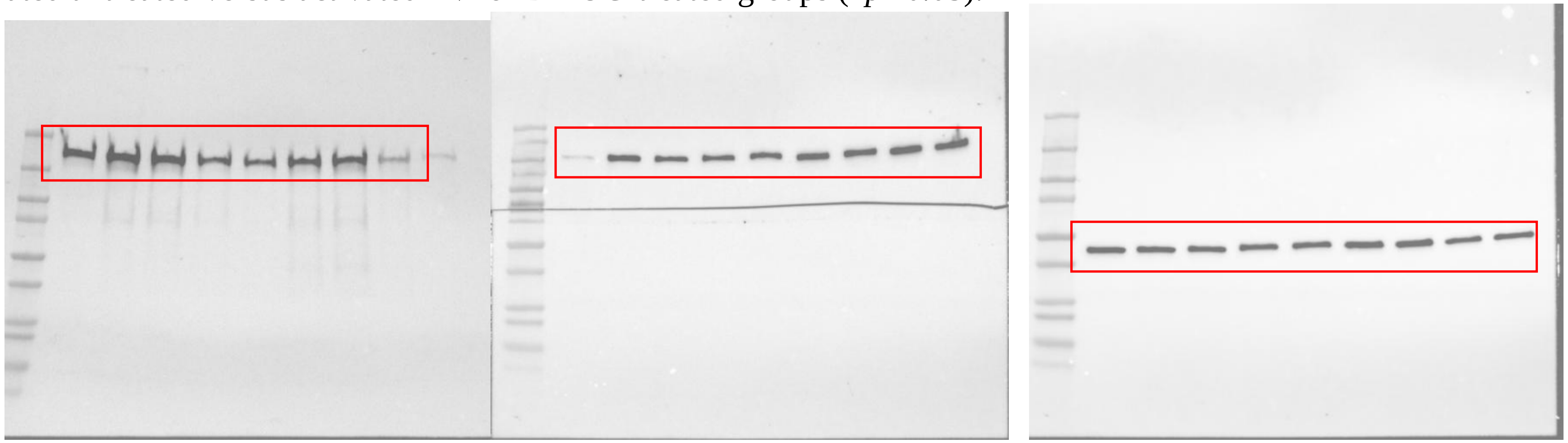

Supplementary material S6: full WB membranes of figure 6

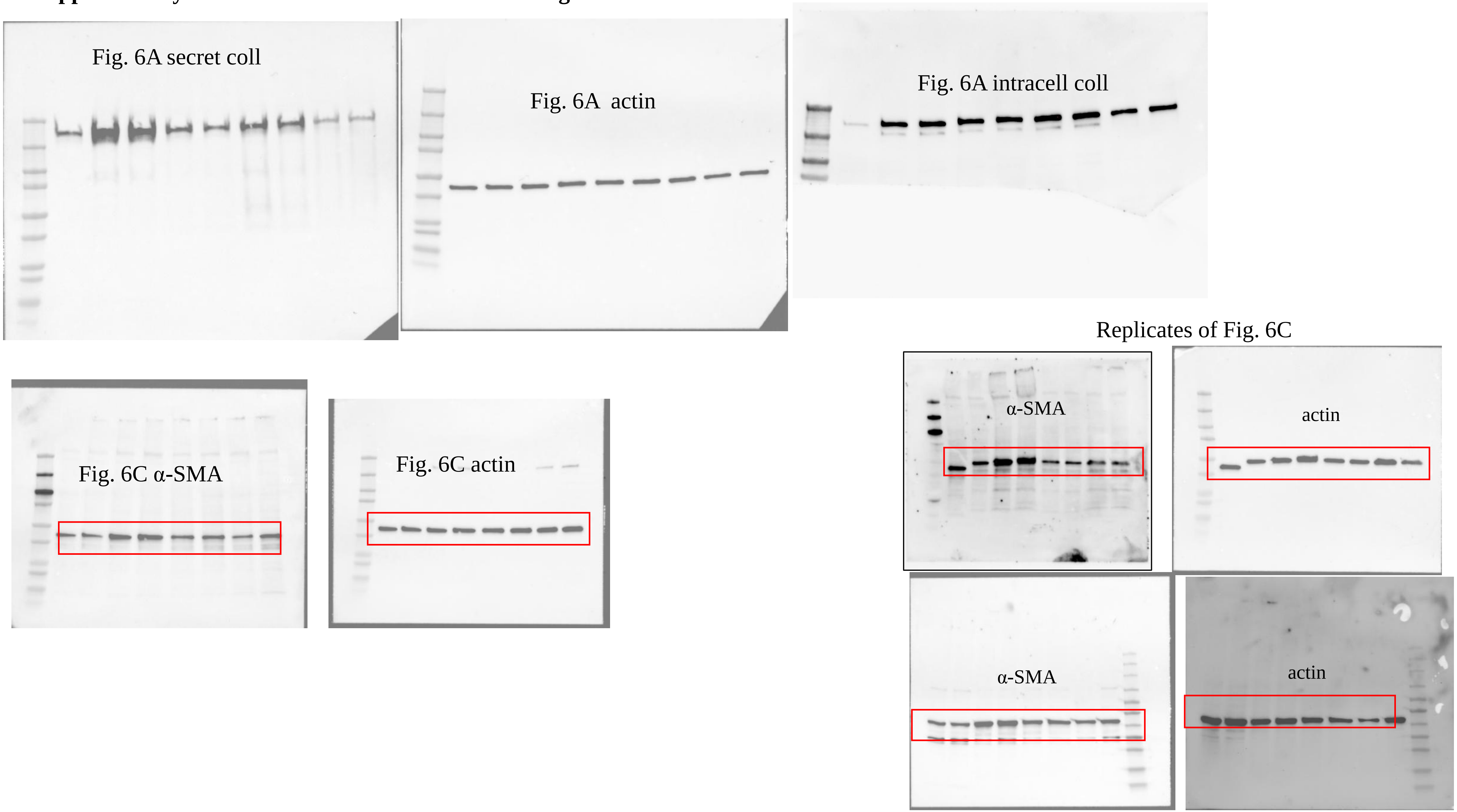

S4

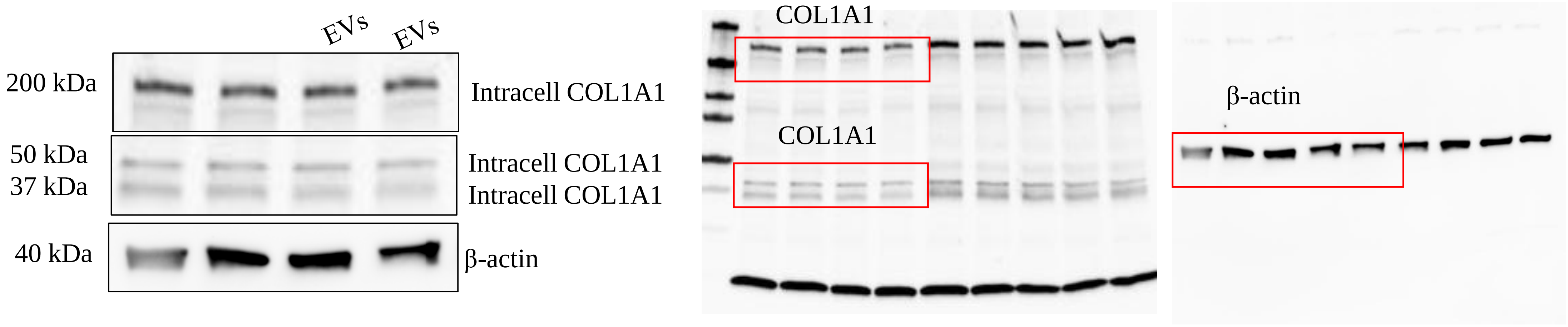

Supplementary figure S4. Assessment of EV protein contamination in non-activated LX-2 cells by Western blot analysis. Cells were either left untreated or treated with 50,000 Brew- or DMEM-derived EVs per cell. Lanes 1 and 2: untreated LX-2 cells; lane 3: LX-2 cells treated with 50,000 Brew-EVs per cell; lane 4: cells treated with 50,000 DMEM-EVs per cell. No differences in collagen levels were observed between untreated and EV-treated LX-2 cells

S5

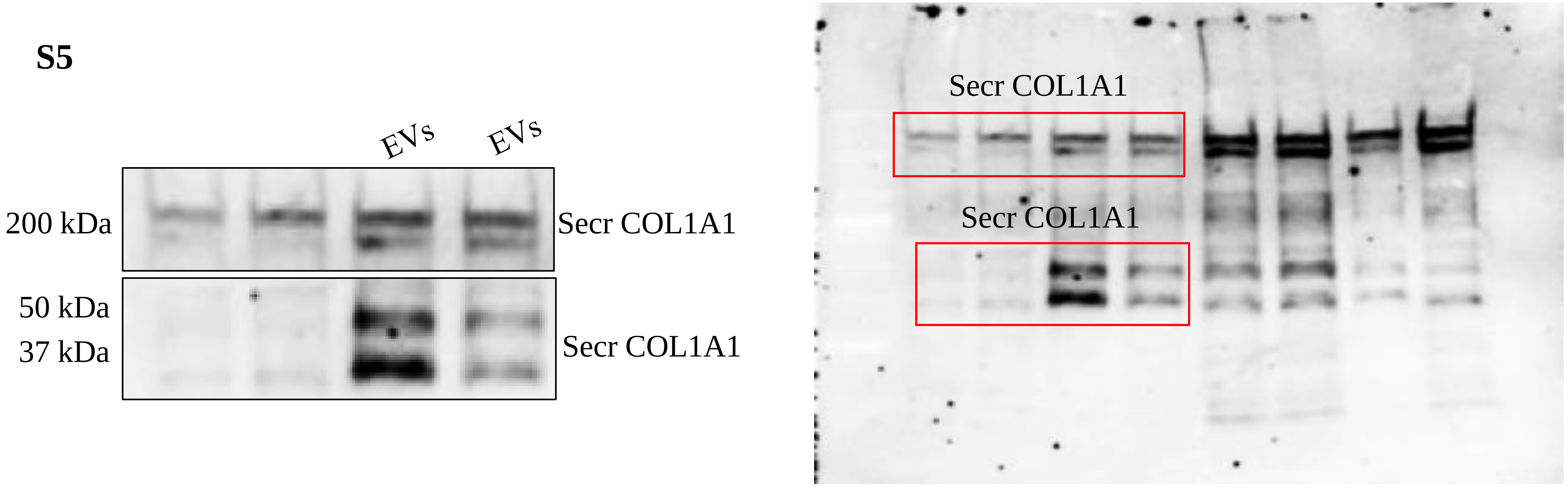

Supplementary figure S5. Assessment of EV protein contamination in TCA-precipitated secretome of non-activated LX-cells by Western blot analysis. Cells were either left untreated (lanes 1 and 2) or treated with 50,000 Brew EVs per cell (lane 3) or 50,000 DMEM-derived EVs per cell (lane 4). Collagen bands of approximately 50 kDa and 37 kDa were detected exclusively in EV-treated LX-2 cells, indicating potential protein contamination associated with the EV preparations. Abbreviations: TCA, trichloroacetic acid; secr, secreted.
